# Mineral Acids in Dysentery

**Published:** 1864-04

**Authors:** James Henderson

**Affiliations:** Medical Officer to the Chinese Mission Hospital, Shanghai


### MINERAL ACIDS IN DYSENTERY.

By Dr. JAMES HENDERSON, Medical Officer to the Chinese Mission Hospital, Shanghai.

[The following is from the Annual Report of the Hospital at Shanghai—a hospital which, from the great number of patients and the variety of the cases treated, must well tax the energies of its sole medical officer, and reflects the greatest credit on his professional skill.]

I found cholera and dysentery prevailing to a great extent and proving very fatal. Soon after the middle of September, I became much struck with the rather unusual type which dysentery in most cases assumed. A few days after the disease commenced, typhoid symptoms usually appeared, great prostration supervened, frequent pulse with a dry brown tongue, and sometimes slight delirium. The whole nervous system seemed prostrated, plainly indicating stimulant treatment, and yet the ordinary stimulants produced no beneficial or decided effect. Wine, brandy, ammonia, camphor, mercury and opium seemed in many cases useless, either to modify the bowel complaint, or rouse the nervous system; indeed, mercury and ammonia seemed to do mischief. Quinine, when retained, appeared to do good, but only in some cases would it remain on the stomach, the mortality was great, and decomposition of the body unusually rapid. On examining the blood of some patients, and comparing it with blood taken from a healthy individual, the former gave a much more decided alkaline reaction than the latter. Even the urine of patients with typhoid symptoms gave in many cases an alkaline reaction, thus indicating a condition of super-alkalinity in the body, or at least, a deficiency of acid. This led me to adopt an acid mode of treatment, which proved decidedly beneficial and successful; and as hydrochloric acid enters so largely into the composition of the tissues of the body, I preferred it to the other acids. It is best to give it with some bitter tonic and a little laudanum, if necessary, every three or four hours, and after the second or third dose, a change for the better is in most cases visible. After adopting this method of treatment at the end of September, very few died, if the treatment was commenced before complete prostration of the nervous system ensued. The patients themselves seemed to feel the good effects of the acid, one man who was admitted in a very weak and prostrate state, with frequent and painful stools, used to call out for more medicine, declaring that it put new life into his body. In about two-thirds of all the cases of dysentery which presented themselves, between the beginning of September and the middle of December, symptoms more or less of a typhoid character were present, and, in a large number they were very marked and decided.

Some cases of pure typhoid fever occurred during the months of November and December, and in the treatment of this disease, the beneficial effects of hydrochloric acid were strikingly manifest. In the treatment of typhoid fever they seem to me as decidedly beneficial and *specific*, as the effects of quinine in ague. Through the "London Medical Times and Gazette" I have recommended a *full* trial of this remedy, in typhoid fever and in dysentery with typhoid symptoms, to be made in the London hospitals. The good effects of hydrochloric acid in typhoid fever are easily explained. Ammonia in large quantities is constantly formed and emitted from the body of a patient with typhoid, and indeed in all cases of low fever. In all cases and conditions, where destructive metamorphosis of the tissues of the body proceeds more rapidly than the nutritive metamorphosis, an excess of ammonia is given off from the body, and the blood is found to be more alkaline than when the functions of the body are in a normal state. The breath even, when analyzed after fasting or great exertion, contains a larger proportion of ammonia than after rest. Moreover, it has been proved by injecting ammonia into the veins of animals, that symptoms are produced exactly similar to those in patients suffering from typhoid fever. Thus clearly indicating that in typhoid fever there is an excess of alkali in the system, in other words, there is not acid present in sufficient quantity to neutralize the alkali, and surely in such a condition a treatment by acids is clearly indicated. The blood of patients suffering from scurvy and purpura, is almost exactly the same as the blood of a patient in typhoid fever. These diseases are universally treated by acids, and why should not also low forms of fever?

In most cases, after the second or third dose, the tongue begins to clean, there is less thirst, the diarrhœa abates, and there is less depression and languor. When there was delirium or low muttering, I gave the acid and bitter infusion in half a wine glass-full of port wine, instead of cold water, which improved all the symptoms even in the most acute cases In many cases of dysentery, ipecacuan wine given with the acid was useful.—*Braithwaite's Retrospect.*

